# Role of non-thermal electrons in ultrafast spin dynamics of ferromagnetic multilayer

**DOI:** 10.1038/s41598-020-63452-3

**Published:** 2020-04-14

**Authors:** Je-Ho Shim, Akbar Ali Syed, Jea-Il Kim, Hong-Guang Piao, Sang-Hyuk Lee, Seung-Young Park, Yeon Suk Choi, Kyung Min Lee, Hyun-Joong Kim, Jong-Ryul Jeong, Jung-Il Hong, Dong Eon Kim, Dong-Hyun Kim

**Affiliations:** 1 Department of Physics and Center for Attosecond Science and Technology, POSTECH, Pohang, 37673 South Korea; 2 Max Planck POSTECH/KOREA Research Initiative, Pohang, 37673 South Korea; 3 Department of Physics, Chungbuk National University, Cheongju, 28644 South Korea; 4 College of Science, China Three Gorges University, Yichang, 443002 P. R. China; 5 Division of Industrial Metrology, Korea Research Institute of Standards and Science, Daejeon, 34113 South Korea; 6 Spin Engineering Physics Team, Korea Basic Science Institute, Daejeon, 34133 South Korea; 7 Department of Material Science and Engineering and Graduate School of Energy Science and Technology, Chungnam National University, Daejeon, 34134 South Korea; 8 Department of Emerging Materials Science, Daegu Gyeongbuk Institute of Science and Technology, Daegu, 42988 South Korea

**Keywords:** Spintronics, Magneto-optics

## Abstract

Understanding of ultrafast spin dynamics is crucial for future spintronic applications. In particular, the role of non-thermal electrons needs further investigation in order to gain a fundamental understanding of photoinduced demagnetization and remagnetization on a femtosecond time scale. We experimentally demonstrate that non-thermal electrons existing in the very early phase of the photoinduced demagnetization process play a key role in governing the overall ultrafast spin dynamics behavior. We simultaneously measured the time-resolved reflectivity (TR-R) and the magneto-optical Kerr effect (TR-MOKE) for a Co/Pt multilayer film. By using an extended three-temperature model (E3TM), the quantitative analysis, including non-thermal electron energy transfer into the subsystem (thermal electron, lattice, and spin), reveals that energy flow from non-thermal electrons plays a decisive role in determining the type I and II photoinduced spin dynamics behavior. Our finding proposes a new mechanism for understanding ultrafast remagnetization dynamics.

## Introduction

The photoinduced ultrafast demagnetization behavior of ferromagnetic systems by a femtosecond laser has attracted considerable attention due to possible applications for future ultrafast spin devices and magnetic information techniques^1–3^. Photoinduced spin dynamics allows us to control magnetic moments on a femtosecond time scale simply by optical pulses^4,5^, as well as by external field or spin current^6,7^. Since the seminal work on a Ni single layer by Beaurepaire *et al*.^8^, numerous studies have investigated the underlying mechanism of ultrafast photoinduced spin dynamics^9–13^. It has been generally accepted that energy from photons is transferred first into the electron sub-system within a few tens of femtoseconds and hot electrons then transfer their energies into the spin and the lattice sub-systems, leading to a final equilibrium state among electron, spin, and lattice sub-systems^8–10^.

**Figure 1:**
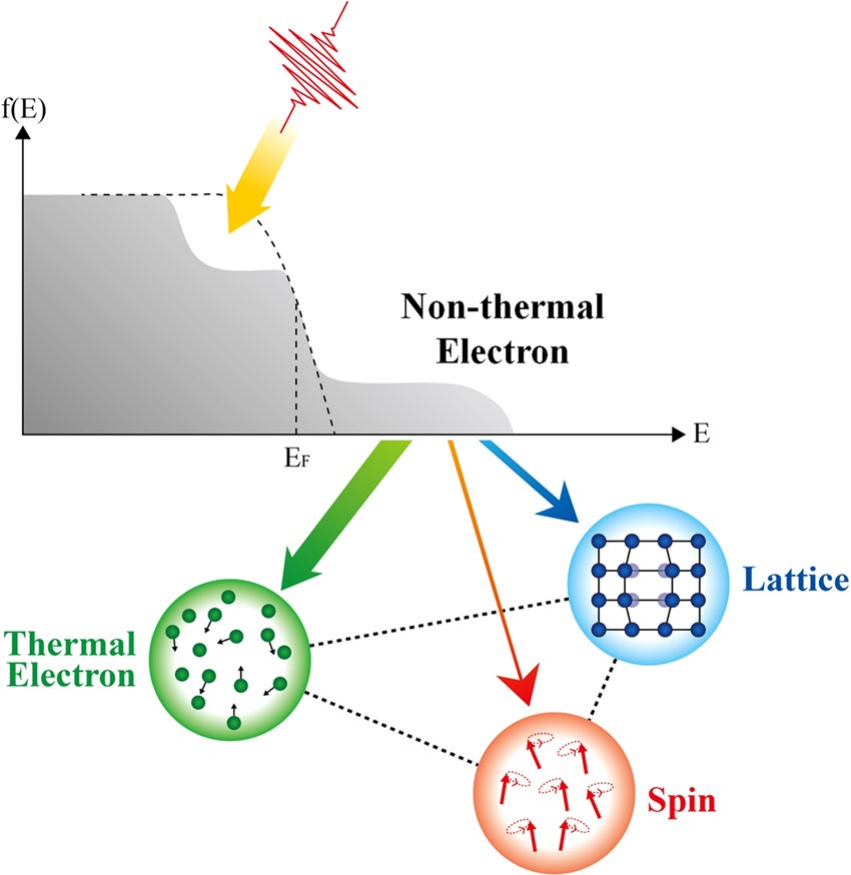
Schematic diagram for energy transfer Non-thermal electrons are excited out of Fermi-Dirac distribution by a laser pulse. Energy transfers from non-thermal electrons to thermal electrons, lattice, and spin sub-systems take place. Thickness of color arrows (green, blue, red) are generalized energy transfer ratio to each system (thermal electrons, lattice, spin) in the low *F*_*p*_ case. Dotted lines are interaction of each system.

Ultrafast spin dynamics triggered by the femtosecond laser is inevitably associated with hot electrons^14–16^. The irradiation of samples with laser pulses induces a change of the electron distribution near the Fermi energy, and further excitation could result in the temporary existence of non-thermal electrons off the thermal Fermi-Dirac distribution^17–21^, as illustrated in Fig. 1. The initial excitation of non-thermal electrons implies that they will be critically involved with energy flow in a very early phase among electron, spin, and lattice sub-systems^22,23^. Although the ultrafast behavior of the non-thermal electrons in normal metals^18,20,24^ and gapped materials^25,26^ has been explored, little is known about the non-thermal electron behavior in ferromagnetic materials^23,27,28^. Moreover, recent reports indicate that the hot electrons can not only contribute to demagnetization but also enhance magnetization on an ultrafast timescale^29^, implying that the understanding and control of non-thermal electron dynamics could be crucial in future ultrafast spin applications.

Here, we have investigated the effect of non-thermal electrons in the early phase of the dynamics and demonstrated that (1) the thermalization of non-thermal electrons is slowed down as pump fluence increases and (2) the energy flow among electrons, spins, and lattices, including non-thermal electrons, naturally explains type I and II remagnetization dynamics. We systematically and simultaneously measured both the time-resolved reflectivity (TR-R) and the time-resolved magneto-optical Kerr effect (TR-MOKE) for Co/Pt multilayer films. Then the extended three-temperature model (E3TM) was used for analysis. The analysis clearly shows that the MOKE signal is rather insensitive to the thermalization of non-thermal electrons, which confirms the necessity of simultaneous measurement ofthe reflectivity.

## Results

**Figure 2:**
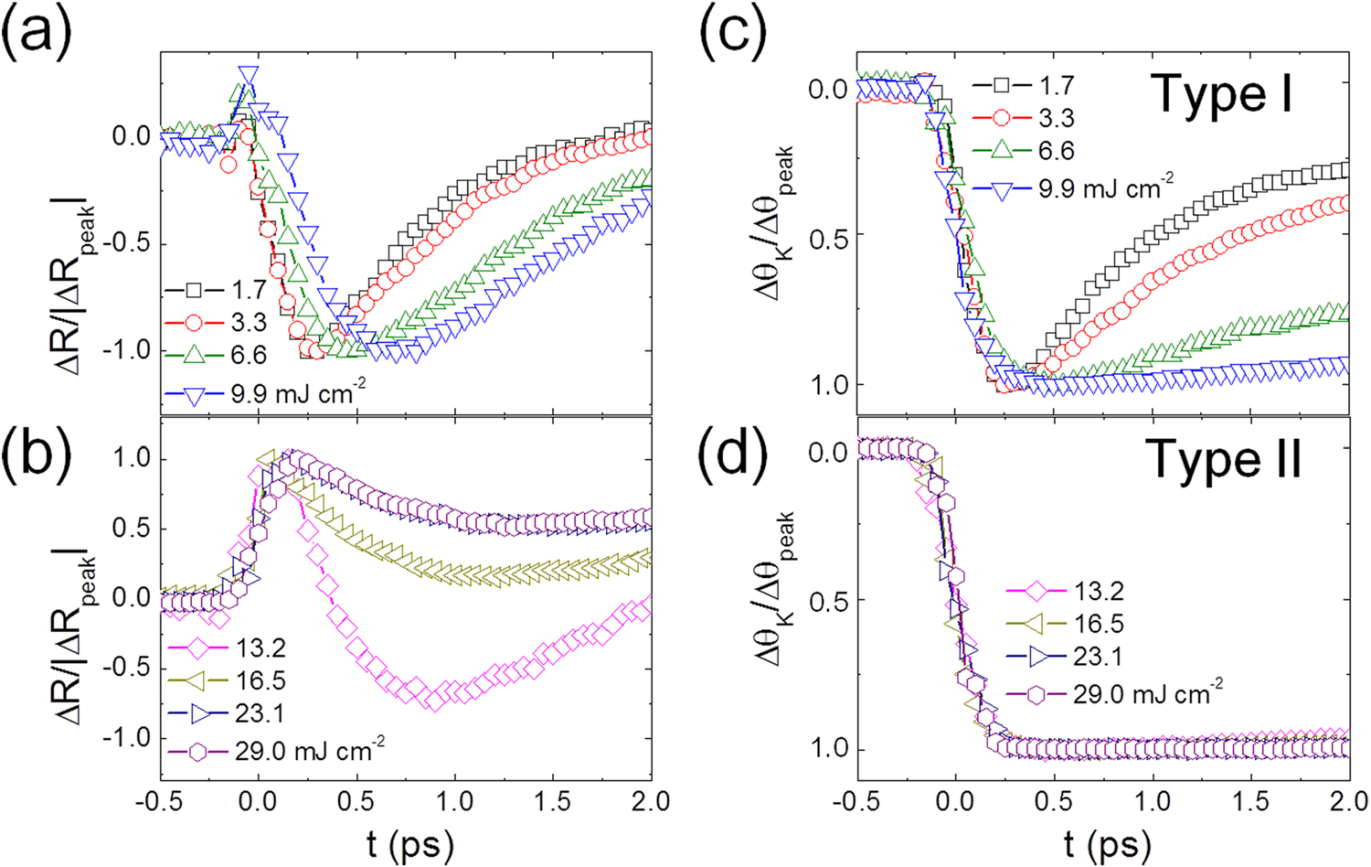
TR-reflectivity measurement and MOKE Measurements. TR-reflectivity measurement (normalized by the maximal change) (**a**) at lower fluences (1.7–9.9 mJ cm^−2^) and (**b**) at higher fluences (13.2–29. mJ cm^−2^). TR-MOKE measurement (normalized by the maximal change) (**c**) at lower fluences (1.7–9.9 mJ cm^−2^), showing type I remagnetization dynamics and (**d**) at higher fluences (13.2–29.0 mJ cm^−2^), showing type II remagnetization dynamics.

TR-R and TR-MOKE were measured for [Co (6.2 Å)/Pt (7.7 Å)]_5_ multilayer film for pump fluences *F*_P_: 1.7 ≤ *F*_P_ ≤ 29.0 mJ cm^−2^. An external magnetic field of 1.7 kOe was applied normal to the film surface. The coercivity and saturation field determined by static magnetic hysteresis were 0.94 and 1.58 kOe, respectively. The change of measured reflectivities (ΔR) was normalized by their peak values for a given laser fluence, as seen in Fig. 2. At lower fluences (Fig. 2(a)), the photoinduced ΔR depends on *F*_P_ such that the reflectivity rapidly decreases, reaching the minimum on a sub-ps time scale, and then is recovered afterward. It is interesting to note that at higher fluences (Fig. 2(b)), ΔR does not exhibit the same simple behavior, as in the case of lower fluences. For example, in the case of *F*_*P*_ = 13.2 mJ cm^−2^, the reflectivity reaches the maximum at t = ~300 fs, but decreases to the minimum at t = ~900 fs, and then relaxes on a longer timescale. The initial abrupt increase of ΔR could be a typical signature of the non-thermal electron excitation, as observed for other noble metals^18,30^. Another possible origin for the nontrivial TR-R trend is a strain effect triggered by laser pulse^31,32^, whereas a timescale of the acoustic wave generated by the laser pulse is on a sub or few tens of picoseconds, which is much longer than the time window (2 ps) in the present study. In supplementary information Note S3, we have discussed coherent phonon oscillation, where the coherent phonon oscillation periods are about 5–10 ps. Therefore, we consider that the nontrivial TR-R behavior is still due to the non-thermal electron excitation above the Fermi energy without being involved with any specific gap structure or strain effect.

The TR-MOKE signal, also normalized by the maximum demagnetization signal, is plotted in Fig. 2(c) (*F*_P_ ≤ 9.9 mJ cm^−2^) and 2(d) (*F*_P_ ≥ 13.2 mJ cm^−2^). In the case of low *F*_P_ (Fig. 2(c)), demagnetization is followed by subsequent remagnetization, where the characteristic remagnetization time gets longer as *F*_P_ increases, which is conventionally categorized as type I remagnetization dynamics^10,14^. For higher *F*_P_ (Fig. 2(d)), the characteristic remagnetization time becomes indefinitely long, indicating the transition to type II remagnetization dynamics. Once *F*_P_ becomes greater than a threshold value of *F*_P_ (13.2 mJ cm^−2^), the normalized TR-MOKE curves are found to fall into the universal one regardless of fluences.

## Discussion

To better understand the dynamics and associated energy flows between electrons, spins, and lattices, we initially used the conventional 3-temperature model (3TM), not considering non-thermal electrons. We failed to fit both TR-R and TR-MOKE experimental data (Supplementary information Note S1 for a more detailed discussion). We then adopted an extended 3TM (E3TM) that considers non-thermal electrons^22,23^. Four coupled differential equations of E3TM are written as

1 $$\begin{array}{c}\frac{dN}{dt}=p[t]-{p}_{e}[t]-{p}_{l}[t]-{p}_{s}[t]\\ {C}_{e}[{T}_{e}]\frac{d{T}_{e}}{dt}={p}_{e}[t]-{G}_{el}({T}_{e}-{T}_{l})-{G}_{es}[{T}_{e},{T}_{s}]({T}_{e}-{T}_{s})\\ {C}_{l}[{T}_{l}]\frac{d{T}_{l}}{dt}={p}_{l}[t]-{G}_{el}({T}_{l}-{T}_{e})-{G}_{ls}({T}_{l}-{T}_{s})-{K}_{l}{({T}_{l}-300)}^{3}\\ {C}_{s}[{T}_{s}]\frac{d{T}_{s}}{dt}={p}_{s}[t]-{G}_{es}[{T}_{e},{T}_{s}]({T}_{s}-{T}_{e})-{G}_{ls}({T}_{s}-{T}_{l})\end{array}$$

2 $$\begin{array}{l}{p}_{i}[t]=\frac{{G}_{ei}}{{C}_{e}}N\\ i=e,l,s\end{array}$$

*N* is the optically pumped, non-thermal electron energy density, and *p*[*t*] is a pump laser source with a Gaussian temporal profile. *p*_*e*_[*t*], *p*_*l*_[*t*], and *p*_*s*_[*t*] represent the energy flows from non-thermal electrons to thermal electrons, lattices, and spin system, respectively, which are defined as in Eq. (2)^18^. *C*_*e*_, *C*_*l*,_ and *C*_*s*_ are the specific heats of the electron, lattice, and spin, respectively. *T*_*e*_, *T*_*l*_, and *T*_*s*_ are the electron, lattice, and spin temperatures, respectively. *G*_*el*_, *G*_*es*_, and *G*_*ls*_ are energy exchange coefficients representing the electron-lattice, electron-spin, and lattice-spin channel, respectively. *G*_*ee*_ is an energy exchange coefficient between non-thermal and thermal electrons. The *K*_*l*_ term describes the thermal diffusion of energy via the lattice, which is modeled to be proportional to the third power of the temperature increase of the lattice system^12,33^.

**Figure 3:**
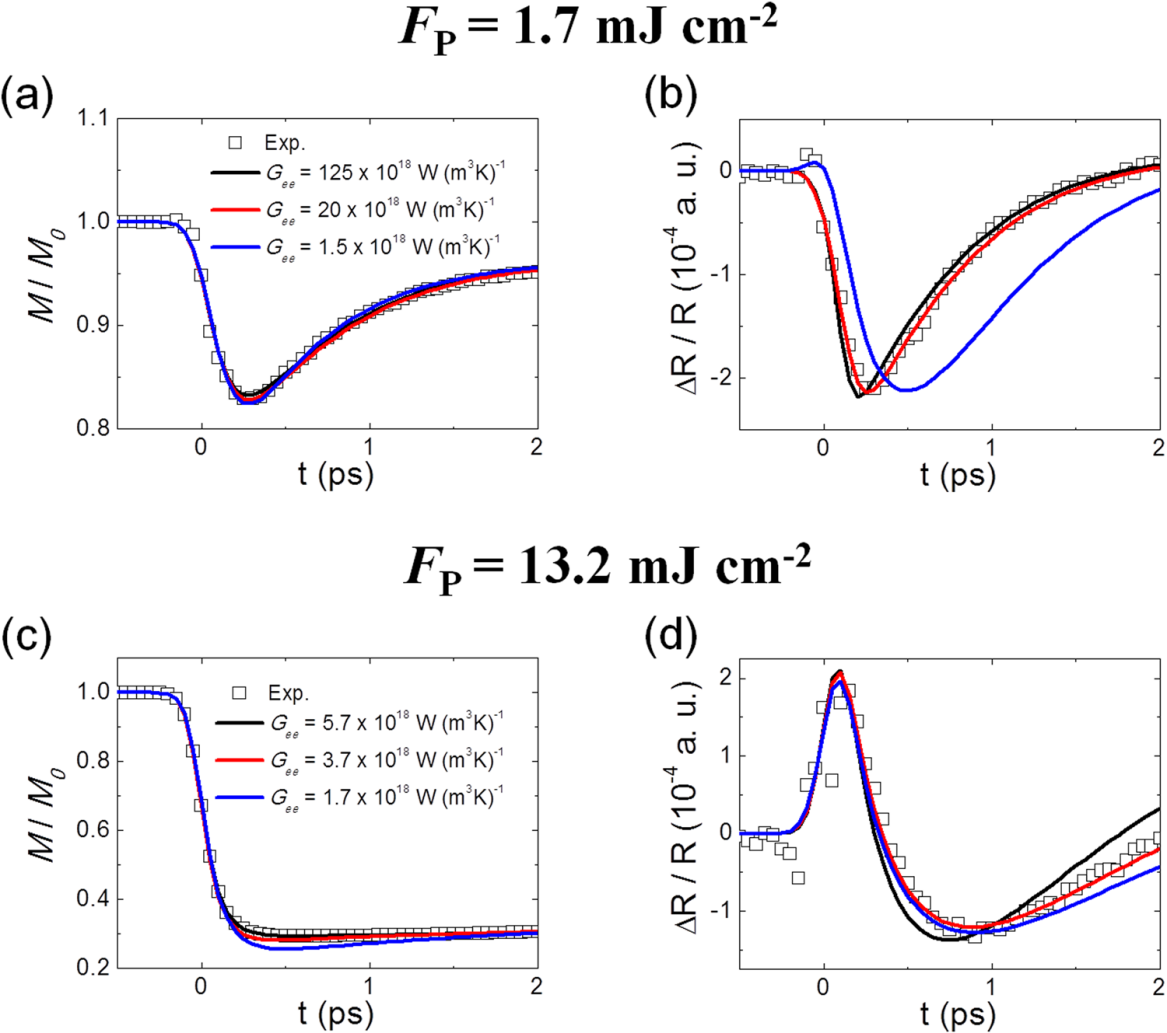
Affect of *G*_*ee*_ fitting parameter. Simultaneous fittings of TR-MOKE (**a,c**) and R (**b,d**) for different *G*_*ee*_ values at *F*_*p*_ = 1.7 mJ cm^−2^ (**a,b**) and *F*_*p*_ = 13.2 mJ cm^−2^ (**c,d**). The fitting clearly shows that *G*_*ee*_ is sensitive to reflectivity but not to MOKE signal.

It turns out that the reflectivity in the early phase depends sensitively on *G*_*ee*_, as shown in Fig. 3. At both low and high fluences, TR-MOKE fitting is not much affected by *G*_*ee*_ (Fig. 3(a,c)), while TR-R fitting is quite sensitive to *G*_*ee*_ (Fig. 3(b,d)). This clearly reveals the critical role of non-thermal electrons and their interactions with thermal electrons and demonstrates that the simultaneous measurement of R and MOKE is crucial in the analysis of energy flow.

**Figure 4:**
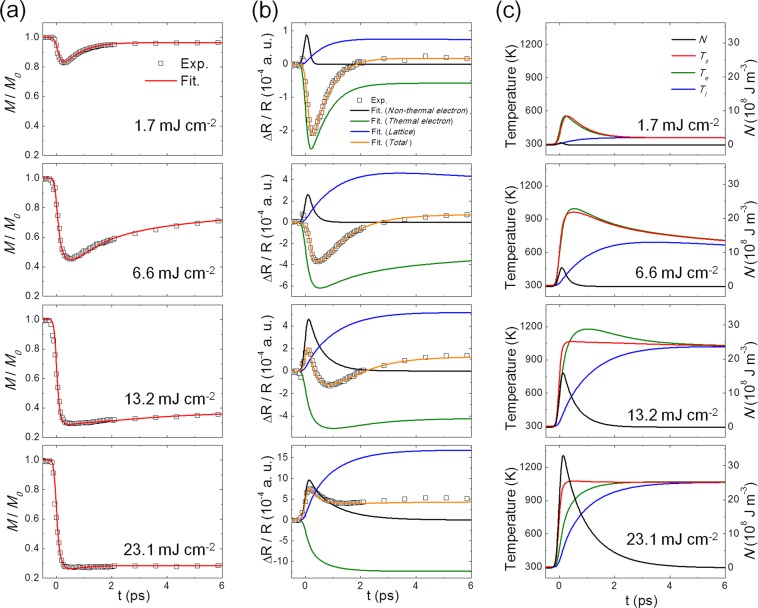
E-3TM fitting results. Fitting of (**a**) TR-MOKE and (**b**) TR-R experimental data (open dots). The contributions from non-thermal electrons (black), thermal electrons (green), and lattice (blue) as well as total sum of them (orange) are plotted. (**c**) Temporal change of extracted Non-thermal electron density (*N*), spin (*T*_*s*_), thermal electron (*T*_*e*_), and lattice (*T*_*l*_) temperature. for the fluences of 1.7, 6.6, 13.2, and 23.1 mJ cm^−2^.

The examples of E3TM fitting are plotted in Fig. 4. In Fig. 4(a), well-fitted TR-MOKE data are plotted for *F*_*P*_ = 1.7, 6.6, 13.2, and 23.1 mJ cm^−2^. TR-R data are also well fitted, as depicted in Fig. 4(b). The contribution to the reflectivity from non-thermal electrons, thermal electrons, and lattice is extracted (Supplementary information Note S1) and plotted in Fig. 4(b). The contribution from thermal electrons (green) exhibits a sharp decrease on a sub-ps time scale, followed by a relatively slow recovery. The subsequent recovery dynamics of thermal electrons are sensitively dependent on *F*_P_ and the reflectivity dip is delayed, which is a direct consequence of heating, particularly in the case of higher *F*_P_.

Very interestingly, there seems to be a non-negligible contribution from non-thermal electrons (black) for all cases of *F*_*P*_. Even for lower pump fluences such as *F*_*P*_ = 1.7 mJ cm^−2^, the non-thermal electrons contribute, although weak. For higher fluence cases such as *F*_*P*_ = 13.2 mJ cm^−2^, the contribution from non-thermal electrons becomes comparable to that from thermal electrons. The contribution from non-thermal electrons exists only in the early phase and rapidly vanishes within ~2 ps under higher fluences. The degree of the non-thermal electron contribution is high, and the decaying time is extended. With lower fluences, the overall reflectivity behavior is mostly determined by thermal electrons (green) and lattice (blue). Positive lattice contribution with much longer recovery time and negative thermal electron contribution with much shorter recovery time are summed, leading to a typical TR-R behavior of rapid decrease, reaching a minimum, and then subsequent recovery.

On the other hand, with higher fluences, the contribution from non-thermal electrons (black) becomes non-negligible, particularly in the early phase (t < 1 ps), leading to a complex TR-R behavior as observed in Fig. 2(b). Additional contribution from the non-thermal electrons in the early phase fits the observed nontrivial TR-R behavior very well, as demonstrated in Fig. 4(b).

In Fig. 4(c), the temperatures, *T*_*s*_ (red), *T*_*e*_ (green), and *T*_*l*_ (blue), together with non-thermal electron energy density *N* (black), are plotted for various *F*_*P*_. For low *F*_*P*_ (1.7 mJ cm^−2^), *T*_*s*_ and *T*_*e*_ exhibit a typical behavior during photoinduced demagnetization^8–10^, where *T*_*s*_ and *T*_*e*_ are almost identical to each other; hence, a simplified 2-temperature model could be valid. However, as *F*_*P*_ increases, the non-thermal electrons influence the temperatures of the other sub-systems. In the case of *F*_*P*_ = 6.6 mJ cm^−2^, *T*_*e*_ is only slightly higher than *T*_*s*_, whereas for *F*_*P*_ = 13.2 mJ cm^−2^, *T*_*e*_ and *T*_*s*_ differ substantially, with *T*_*s*_ arising slightly faster than *T*_*e*_, although the maximum temperature of *T*_*e*_ is still higher than that of *T*_*s*_. The quicker increase of *T*_*s*_ in the early phase is directly linked to the role of non-thermal electrons since non-thermal electrons take the photon energy first and then redistribute the energy to thermal electrons, spin, and lattice sub-systems, as in Eq. (1). Therefore, the observed behavior of *T*_*e*_ and *T*_*s*_ implies that *G*_*ee*_ significantly changes under high fluences. In the case of *F*_*P*_ = 23.1 mJ cm^−2^, the temperature discrepancy between *T*_*e*_ and *T*_*s*_ becomes even bigger due to the more considerable influence of the non-thermal electrons. Within 1 ps, non-thermal electrons absorb most of the photon energy and distribute it into spin, thermal electrons, and lattice. It should be noted that *T*_*s*_ increases faster than *T*_*e*_, implying further modification of the energy exchange coefficients. At higher fluences (13.2 and 23.1 mJ cm^−2^), *T*_*s*_ rapidly approaches *T*_*C*_ (1131 K) and exhibits a very slow decrease afterward, where the rapid increase of *T*_*s*_ is dominated by the transferred energy from the non-thermal electrons. The maximum *T*_*s*_ is less than *T*_*C*_ in all the cases, and hence, the application of E3TM remains valid. As seen in Fig. 4(a), at the fluence of 23.1 mJ cm^−2^, the TR-MOKE experiment data indicate the maximum demagnetization of 76%, which reveals the continued presence of significant magnetic ordering even at this high fluence, which seems to be consistent with the consideration that the non-thermal electrons take more energy as *F*_*P*_ increases so that the spin system does not become fully demagnetized. While *T*_*s*_ approaches *T*_*C*_, thermal electrons and lattice still receive energy from non-thermal electrons and the excessive energy in thermal electrons and lattice continues to interact with the spin sub-system, which could effectively lead to the slow recovery of the spin system.

**Figure 5:**
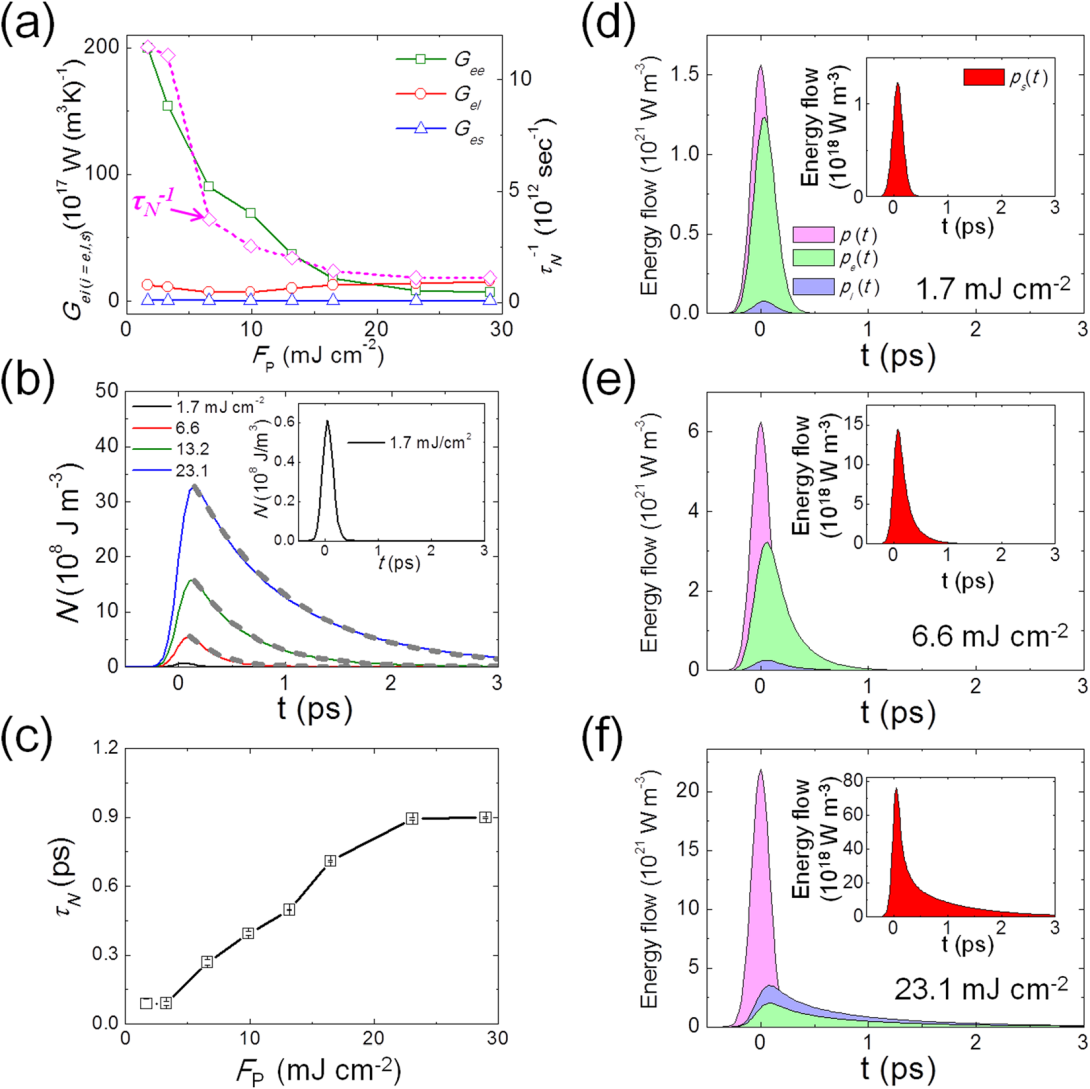
E-3TM fitting analysis. (**a**) Variation of energy exchange coefficients of *G*_*ee*_, *G*_*el*_, and *G*_*es*_ with respect to the fluences (*F*_*p*_). The plot of *τ*_*N*_^−1^ (dotted line) exhibiting the same behavior with respect to *F*_*p*_, where *τ*_*N*_ is the decay time of non-thermal electron population as found in (**b**). (**b**) Variation of non-thermal electron energy density (*N*) with respect to the fluence (*F*_*p*_ = 1.7, 6,6, 13.2, 23.1 mJ cm^−2^). The inset is a zoomed-in plot for *F*_*p*_ = 1.7 mJ cm^−2^. The dotted curves are fits by exponential function to extract the decay time (*τ*_*N*_) (**c**) Variation of decay time *τ*_*N*_ with respect to *F*_*p*_. Temporal change of *p*[*t*], *p*_*e*_[*t*]*, p*_*l*_[*t*] for (**d**) *F*_*p*_ = 1.7, (**e**) 6.6, and (**f**) 23.1 mJ cm^−2^. Inset Fig.s are zoomed-in *p*_*s*_[*t*] for each of *F*_*p*_.

These fitting results reveal how the energy exchange coefficients between sub-systems change. Figure 5(a) shows the change of *G*_*ee*_, *G*_*el*,_ and *G*_*es*_ with respect to the fluences. *G*_*es*_ in all the cases is in the order of about 10^16^ W (m^3^K)^−1^, substantially less than *G*_*el*_, which is in the order of 10^18^ W (m^3^K)^−1^ for all fluences; on the other hand, *G*_*ee*_ exhibits the significant variation. For *F*_*P*_ = 1.7 mJ cm^−2^, *G*_*ee*_ ~ 2 × 10^20^ W (m^3^K)^−1^, which is the largest among all the energy exchange coefficients. *G*_*ee*_ remains dominant in low *F*_*P*_ cases (*F*_*P*_ < 9.9 mJ cm^−2^). The larger *G*_*ee*_ should lead to the faster relaxation of energy from non-thermal electrons to thermal ones. However, at lower fluences, the non-thermal electron density is substantially low, as seen in Fig. 4(c); hence, the contribution of non-thermal electrons is negligible. *G*_*ee*_ significantly decreases as *F*_*P*_ increases and becomes comparable to *G*_*el*_ for *F*_*P*_ = 13.2 and 16.5 mJ cm^−2^, and even smaller than *G*_*el*_ for *F*_*P*_ = 23.1 and 29.0 mJ cm^−2^. Along with the reduction of *G*_*ee*_, the effective non-thermal electron density dramatically increases, as shown in Fig. 4(c); hence, the non-thermal electrons play a significant role in the overall spin dynamics. It should be mentioned that the total effective energy exchange coefficients get smaller under higher fluences, leading to a longer remagnetization time, as observed in the experiment.

In Fig. 5(b), the non-thermal electron energy density (*N*) for various fluences is plotted with respect to time. It is clearly observed that the *N* becomes more abundant with higher fluences and the overall shape is elongated with time at higher fluences. In the inset figure at *F*_*P*_ = 1.7 mJ cm^−2^, *N* sharply increases initially and then decreases in the very early phase. The decreasing part of *N* is fitted by an exponential decay (gray dotted line) to determine the characteristic decay time *τ*_*N*_, which is then plotted with respect to *F*_*P*_ in Fig. 5(c); *τ*_*N*_ and *F*_*P*_ exhibit a clear proportionality. The increase of *τ*_*N*_ for higher fluences is consistent with the observed decrease of *G*_*ee*_ in Fig. 5(a). The inverse of *τ*_*N*_ (*τ*_*N*_^−1^) is also plotted in Fig. 5(a), and shows the same fluence-dependent trend as observed for *G*_*ee*_. This confirms that *τ*_*N*_ is effectively inversely proportional to *G*_*ee*_^18^. *τ*_*N*_ can be written as ${ < \tau }_{{\rm{N}}} > =\frac{1}{\langle {{\rm{\nu }}}^{{ee}}\rangle }=\frac{\int \Delta {\rm{f}}({\rm{E}}){{\rm{\tau }}}_{{\rm{ee}}}({\rm{E}}){\rm{dE}}}{\int \Delta {\rm{f}}({\rm{E}}){\rm{dE}}}$ averaged over the distribution function. *∆f(E)* is a deviation from Fermi distribution, representing the excitation of electrons and $\langle {{\rm{\nu }}}^{{ee}}\rangle$ the electron-electron collision rate. *∆f(E)* has been typically modeled to be uniform around the Fermi energy^18,34^. However, recently, a non-uniform excited distribution *∆f(E)* has been reported for Au slabs^35^ and Au nanoparticles^34^, where more excitation near the Fermi energy is implied. The relatively slower timescale of excited electrons near the Fermi energy might lead to a slower timescale of ${ < \tau }_{N} >$. Moreover, based on Fermi liquid theory, the electron-electron collision rate follows as^36,37^ $\langle {{\rm{\nu }}}^{{\rm{ee}}}({\rm{T}},{\rm{\omega }})\rangle ={{{\rm{\nu }}}^{{\rm{ee}}}}_{0}({\rm{T}},{\rm{\omega }})\left[1+{\left(\frac{{\rm{\hslash }}{\rm{\omega }}}{2{\rm{\pi }}{\rm{kT}}}\right)}^{2}\right]$, where ${{{\rm{\nu }}}^{{\rm{ee}}}}_{0}({\rm{T}},{\rm{\omega }})$ is the corresponding classical collision frequency proportional to *T*^2^. In the cases where the 2^nd^ term can be neglected, $\langle {{\rm{\nu }}}^{{ee}}\rangle$ is proportional to *T *^2^. In our case, the pump photon energy of 1.5 eV is significantly larger than *kT* so that ${\left(\frac{{\rm{\hslash }}{\rm{\omega }}}{2{\rm{\pi }}{\rm{kT}}}\right)}^{2}$ ≫ 1 and $\langle {{\rm{\nu }}}^{{ee}}\rangle$ is more or less constant implying that the thermalization of non-thermal electron decay time might take longer. Very recently, B. K. Nayak *et al*. has reported the experimental observation of a slower thermalization with increasing pump fluence^38^, similar to our observation. On the other hand, in plasma physics, where hot electrons move freely, the electron-electron collision has been well known to behave as *T*^*−3/2*^, which has been called as Spitzer resistivity^39^. The hot electrons in the conduction band of metal have a similarity to those in high-temperature plasma in a sense that they are free to move around but yet are different in a sense that the density is much higher and so electron-electron correlation effect might be larger. Hence, the hot electrons in the conduction band of metal may show the same trend as in high-temperature plasma but with different scaling, as shown in Ref. ^38^.

In Fig. 5(d) to 5(f), the energy flow of *p*_*e*_[*t*], *p*_*l*_[*t*], *p*_*s*_[*t*], and the laser energy profile *p*[*t*] are plotted. With the increase of *F*_*P*_, *p*_*e*_[*t*] and *p*_*l*_[*t*] become smaller and broader compared to *p*[*t*]. The inset shows that with the increase of *F*_*P*_, *p*_*s*_[*t*] increases, gets sharper, and develops a tail unlike *p*_*e*_[*t*] and *p*_*l*_[*t*]. The peak position of *p*_*s*_[*t*] is around t ~300 fs, which coincides with the temporal moment of the maximal demagnetization time regardless of the fluences in the TR-MOKE data (Fig. 2(c,d)). Further details of *p*_*s*_[*t*] are described in Supplementary information Note S2^40^. An interesting development of the tail in *p*_*s*_[*t*], lasting up to 2 ps in the case of high fluences, originates from the broadening non-thermal electron energy density *N* since *p*_*s*_[*t*] is proportional to the product of *N* and *G*_*es*_, as shown in Fig. S3 of Supplementary information Note S2.

**Figure 6:**
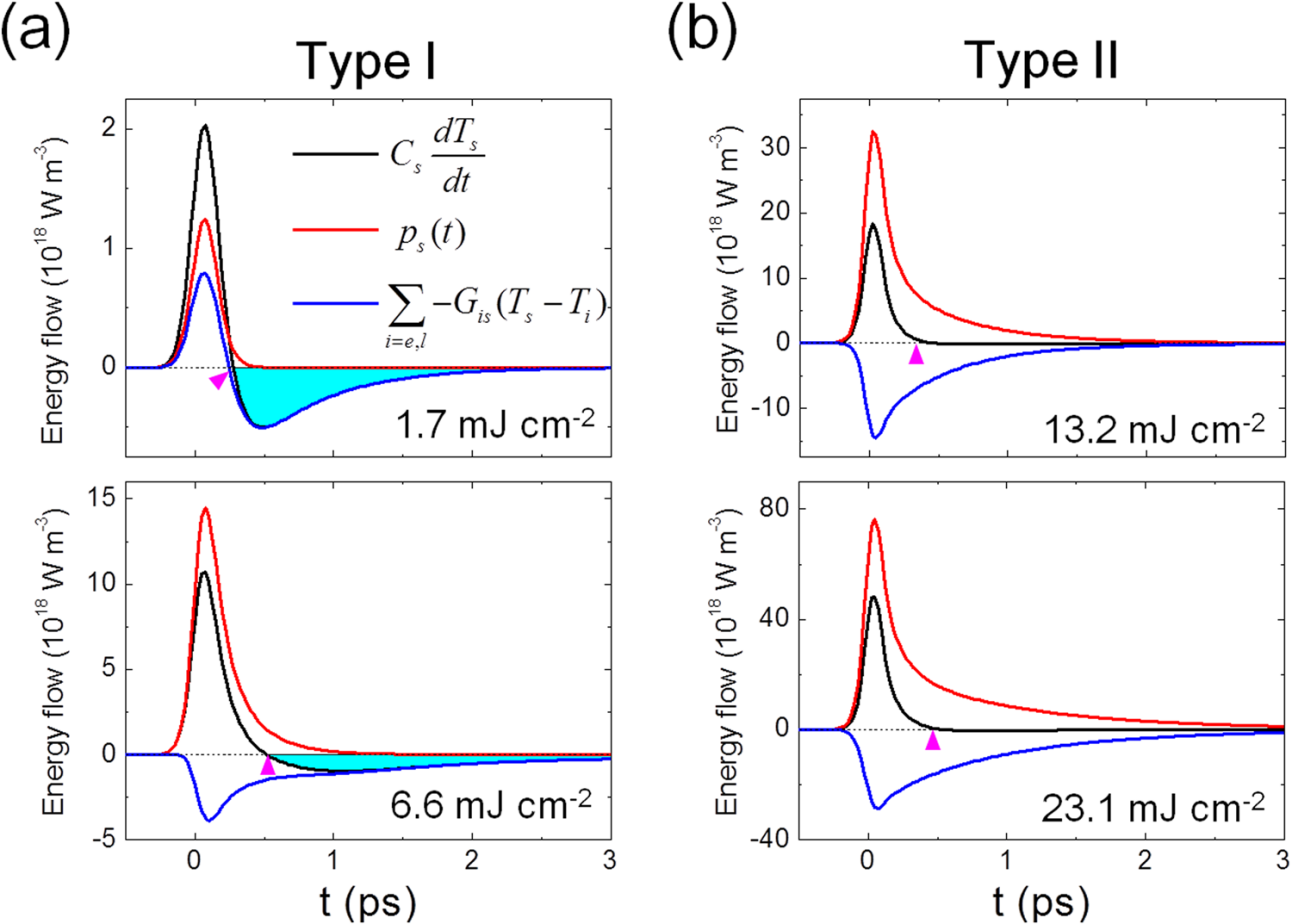
Energy flow in spin system. Comparisons of energy flow terms: ${C}_{s}\frac{d{T}_{s}}{{dt}}$*, p*_*s*_[*t*], and $\sum _{i=e,l}-{G}_{is}({T}_{s}-{T}_{i})$. (**a**) type I remagnetization dynamics (*F*_*p*_ = 1.7 and 6.6 mJ cm^−2^) and (**b**) type II remagnetization dynamics (*F*_*p*_ = 13.2 and 23.1 mJ cm^−2^). The pink arrows represent when the net energy flow into and out of the spin sub-system becomes zero. The blue shaded region is where the net energy flow becomes negative, meaning that the spin sub-system loses its energy.

Such an analysis of energy flow reveals, the energetics of type I and II remagnetization dynamics in detail. In Fig. 6, the energy terms of the spin subsystem in the 4^th^ equation of Eq. (1) are plotted for the early phase of ultrafast photoinduced demagnetization in Fig. 6. As discussed in Fig. 2, the type I (Fig. 2(a)) and type II (Fig. 2(b)) remagnetization dynamics are categorized based on the TR-MOKE behavior. In the case of the low fluence (1.7 mJ cm^−2^), ${C}_{s}\frac{d{T}_{s}}{{dt}}$ (net energy of the spin sub-system) shows a sharp increase boosted by *p*_*s*_[*t*] (the energy flow from non-thermal electrons), and $\sum _{i=e,l}-{G}_{is}({T}_{s}-{T}_{i})$ (the sum of interaction energy flows involved with other sub-systems). However, later, ${C}_{s}\frac{d{T}_{s}}{{dt}}$ becomes negative, due to the change of the sign of $\sum _{i=e,l}-{G}_{is}({T}_{s}-{T}_{i})$, letting the net energy flow out of the spin sub-system. The resulting rapid cooling of the spin by the net flow out of the spin system corresponds to the observed rapid recovery of the TR-MOKE signal, resulting in the type I behavior. A similar trend occurs at *F*_*P*_ = 6.6 mJ cm^−2^. The negative $\sum _{i=e,l}-{G}_{is}({T}_{s}-{T}_{i})$ contribution implies that the energy flows from the spin sub-system to other sub-systems after all. The same behavior is observed for the case of *F*_*P*_ = 6.6 mJ cm^−2^. As seen in the *T*_*s*_ profile in Fig. 4(c), the spin subsystem is excited fast in the early phase due to its much lower heat capacity than that of the thermal electrons and lattice, so that the net energy can flow into other sub-systems (the negative value of $\sum _{i=e,l}-{G}_{is}({T}_{s}-{T}_{i})$, as seen in Fig. 6(a)). The fact that ${C}_{s}\frac{d{T}_{s}}{{dt}}$ becomes negative indicates the fast recovery of the spin sub-system (type I remagnetization dynamics).

On the other hand, at higher fluences of 13.1 and 23.1 mJ cm^−2^ (Fig. 6(b)), $\sum _{i=e,l}-{G}_{is}({T}_{s}-{T}_{i})$ contribution remains negative; however, *p*_*s*_[*t*] is strong and has a positive tail, which can cancel out so that ${C}_{s}\frac{d{T}_{s}}{{dt}}$ remains nearly zero through the later stage of the ultrafast remagnetization process, leading to the slow remagnetization behavior (type II remagnetization dynamics). These results demonstrate that non-thermal electrons play a significant role in determining the overall ultrafast spin dynamics, particularly in determining the type of remagnetization dynamics (type I or II). With respect to the cause of different remagnetization dynamics, although a few mechanisms involved with Elliot-Yafet scattering^10,41–43^ and spin current^6,7,11,44^ have been discussed. Very recently, a quantitative study and comparison between fluence-dependent experiments and a model that includes non-equilibrium effects microscopically has been reported^45^. This investigation revealed that the non-thermal electron contribution could be another critical mechanism in understanding ultrafast photoinduced spin dynamics.

**Figure 7:**
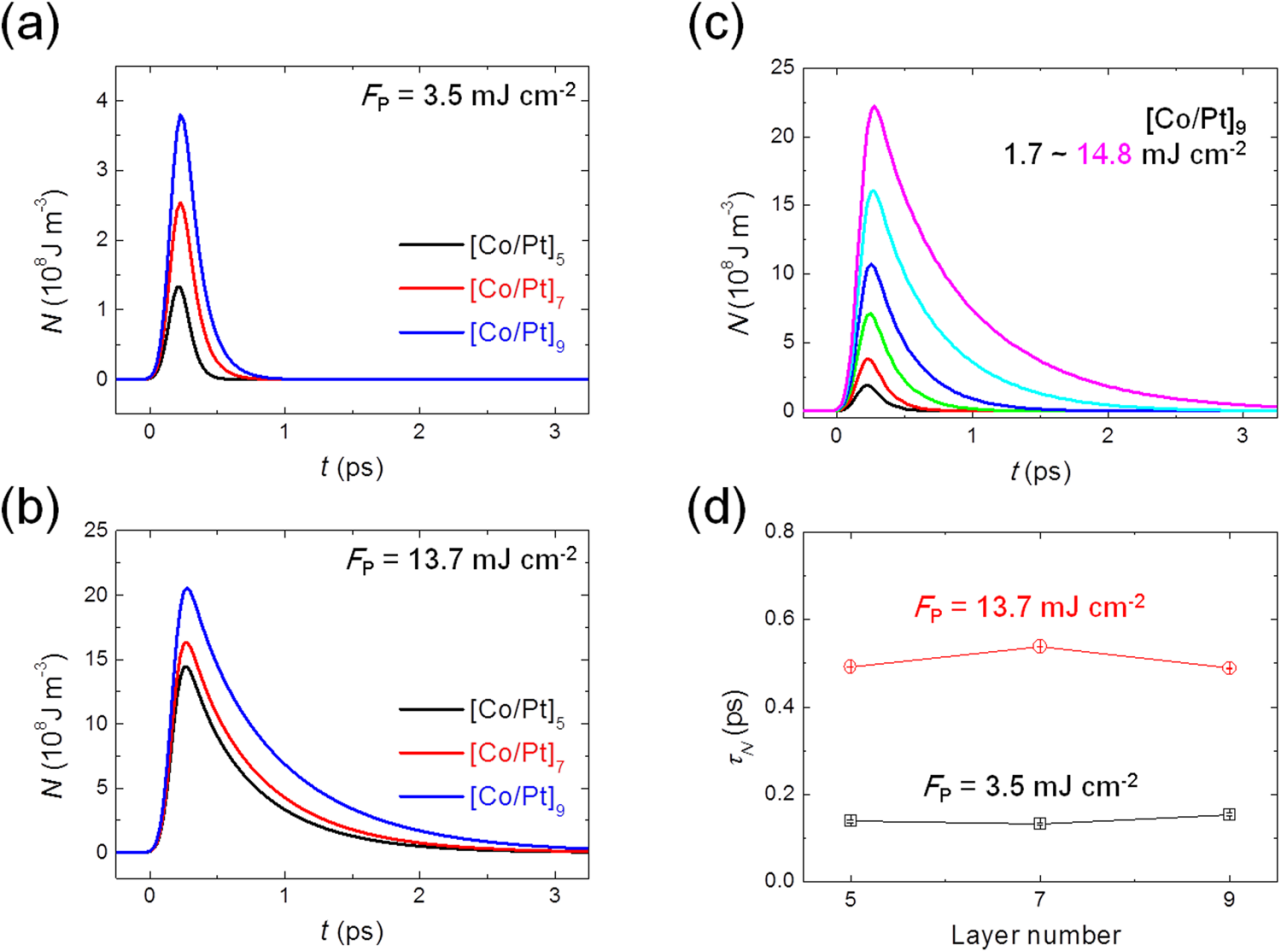
*N* vs. *t* for [Co/Pt]_5_, [Co/Pt]_7_, and [Co/Pt]_9_ multilayers at *F*_P_ = (**a**) 3.5 and (**b**) 13.7 mJ cm^−2^. (**c**) *F*_P_ dependent *N* vs. *t* for [Co/Pt]_9_ multilayer. (**d**) Comparison of non-thermal electron timescale (*τ*_*N*_) for 3 films at *F*_P_ = 3.5 and 13.7 mJ cm^−2^.

Lastly, a strain wave effect is considered in a more systematic way. Although the timescale of the acoustic phonon oscillation is found to be about 5 –10 ps, as described in the Results and Supplementary Information (S3), there might still be a possibility to have a strain wave with modifying the observed TR-Reflectivity and TR-MOKE signal. If any, strain waves will be launched from the surface, respectively from the interface to the substrate with the maximum strength of the strain when the acoustic waves meet in the middle. We have carried out a more careful analysis to exclude the transient strain by carrying out thickness-dependent experiments. We have varied repeat number n of [Co/Pt]_n_ multilayer (n = 5, 7, and 9) so that the total thickness is varied from 8.5 to 15.3 nm. Time-resolved non-thermal electron density (*N*) at various fluences is plotted for 3 thickness cases. In Fig. 7(a), at the fluence of 3.5 mJ cm^−2^, *N* increases as the total thickness increases, since more photon energy is absorbed for thicker samples. The same is observed at the fluence of 13.7 mJ cm^−2^ as in Fig. 7(b). For the thickest sample (n = 9), increases with respect to the fluence, similar to the case of n = 5, as in Fig. 7(c). It should be noted that the timescale of *τ*_*N*_ remains almost the same regardless of the total film thickness at each fluence (Fig. 7(d)), indicating that the strain wave effect can be neglected in our analysis.

## Conclusion

In summary, we have systematically investigated the role of non-thermal electrons in ultrafast photoinduced demagnetization/remagnetization dynamics for a Co/Pt multilayer films and demonstrated that non-thermal electrons play a crucial role in understanding type I and II remagnetization dynamics. Using E3TM, which also considers the contribution from non-thermal electrons in addition to thermal electrons spin and lattice, the excellent fittings to the experimental TR-MOKE and TR-R data reveal the full details of energy transfers involved among sub-systems. In competition with other energy exchange channels, the energy exchange channel of non-thermal electrons, which have been so far neglected, play a crucial role, particularly in the case of high pump fluences. Our findings support a new possible mechanism for explaining the ultrafast spin dynamics behavior.

## Method

### **Time-resolved MOKE/reflectivity measurement**

TR-MOKE and reflectivity measurements with a pump-probe stroboscope were performed on a Co/Pt multilayer. The pump pulses were generated by a Ti: sapphire multipass amplifier operating at a repetition rate of 3 kHz with a center wavelength of 780 nm and a pulse duration of 25 fs. The probe pulses with the same wavelength were generated by a beam splitter. Another beam splitter was placed in the probe beam path, before it was reflected from the sample, to obtain the reference beam and probe pulses for TR-R measurements. Our experimental setup is a polar TR-MOKE setup. The pump beam was focused on the sample along the normal direction (z-axis). The angle between the pump and probe beam was set to 35°. The sample plane is xy-plane, the optical plane is zx-plane, and the magnetization direction is along the z-axis. The incident pump fluence (*F*_P_) was varied from 1.7 to 28.5 mJ cm^−2^ at a fixed probe fluence of 0.3 mJ cm^−2^. A mechanical delay line was implemented in the pump-beam line. A Wollaston polarizer was positioned in front of the two photodiodes to split out the s- and p-polarization components. The resulting measurement generates a difference between the s- and p-polarization components of the probe pulses, as modified by the TR-MOKE at the reflection off a film surface. For the TR-MOKE measurements, the pump beam was modulated using a mechanical chopper at 500 Hz. An external magnetic field of 1.7 kOe normal to the sample was applied throughout the measurements to keep the initial sample condition saturated before the subsequent pump pulse.

### Samples

[Co(6.2 Å)/Pt(7.7 Å)]_5_ multilayer films were deposited by dc magnetron sputtering on Si substrates, then capped by a 22-Å Pt layer to prevent surface oxidation. The structure of the Co/Pt multilayer films with well-defined interfaces was confirmed by a low angle X-ray diffraction and extended X-ray absorption fine structure analysis. The films exhibited perpendicular magnetic anisotropy (*K* = 0.63 MJ m^−3^), and saturation magnetization (*M*_*s*_ = 1.04 × 10^3^ kA m^−1^), which were measured by the Electro-Magnetic Property Measurement System developed in the Korea Basic Science Institute and were confirmed to be similar to literature values^46–48^.

## Supplementary information

Supplementary information.
