# Supplementary material for: Role of non-thermal electrons in ultrafast spin dynamics of ferromagnetic multilayer
Source: Sci Rep. 2020 Apr 14;10:6355. doi: 10.1038/s41598-020-63452-3 (PMC7156415; doi:10.1038/s41598-020-63452-3)
Supplement: Supplementary file 1 — Supplementary information. [file 41598_2020_63452_MOESM1_ESM.docx]

Supplementary information for

Role of non-thermal electrons in ultrafast spin dynamics of ferromagnetic multilayer

**Je-Ho Shim^1,2^, Akbar Ali Syed^1,2^, Jea-Il Kim^1,2^, Hong-Guang Piao^3,4^, Sang-Hyuk Lee^3,5^,** **Seung-Young Park****^6^, Yeon Suk Choi^6^, Kyung Min Lee^7^, Hyun-Joong Kim^8^, Jong-Ryul Jeong^7^, Jung-Il Hong^8^, Dong Eon Kim^1,2a)^, and Dong-Hyun Kim^3b)^**

*^1^Department of Physics and Center for Attosecond Science and Technology, POSTECH, Pohang 37673, South Korea*

*^2^Max Planck POSTECH/KOREA Research Initiative, Pohang 37673, South Korea*

*^3^Department of Physics, Chungbuk National University, Cheongju 28644, South Korea*

*^4^College of Science, China Three Gorges University, Yichang 443002, P. R. China*

*^5^Division of Industrial Metrology, Korea Research Institute of Standards and Science, Daejeon 34113, South Korea*

*^6^Spin Engineering Physics Team,* *Korea Basic Science Institute, Daejeon 34133, South Korea*

*^7^Department of Material Science and Engineering and Graduate School of Energy Science and Technology, Chungnam National University, Daejeon 34134, South Korea*

*^8^Department of Emerging Materials Science, Daegu Gyeongbuk Institute of Science and Technology, Daegu 42988, South Korea*

**S1. Extended three temperature model (E3TM) fitting process**

In order to investigate the fluence-dependent ultrafast dynamics of electrons and spin, we first applied a conventional 3TM to fit the TR-MOKE signals and reflectivity with the following equations:

$\begin{matrix} C_{e}[T_{e}]\frac{dT_{e}}{dt}=-G_{el}\times(T_{e}-T_{l})-G_{es}\left[T_{e},T_{s}\right]\times(T_{e}-T_{s})+P(t) \\ C_{l}[T_{l}]\frac{dT_{l}}{dt}=-G_{el}\times(T_{l}-T_{e})-G_{ls}\times(T_{l}-T_{s})-K_{l}\times(T_{l}-300)^{3} \\ C_{s}[T_{s}]\frac{dT_{s}}{dt}=-G_{es}\left[T_{e},T_{s}\right]\times(T_{s}-T_{e})-G_{ls}\times(T_{s}-T_{l}) \end{matrix}$ (S1),

where *G_el_*, *G_es_*, and *G_ls_* are the electron-lattice, electron-spin, and lattice-spin energy interaction coefficient, respectively. From the TR-MOKE data, the spin temperature was estimated based on the relation of *M* ∝ (1 – (*T*_s_/*T*_C_))^0.5^, where *T*_C_ is the Curie temperature (1131 K for Co).

The reflectivity was first fitted based on the following relation,

$\frac{\Delta R}{R}\sim a\Delta T_{e}+b\Delta T_{l}$ (S2),

where *a* and *b* are fitting parameters, and *∆T_e_* and *∆T_l_* are the change of electron and lattice temperature, respectively, induced by the laser pulse. First, we start fitting for the cases of low *F*_P_ because *T_e_* and *T_s_* are expected to be very close. While fitting reflectivity, we also simultaneously consider the TR-MOKE signal.

Fig. S1 shows the best fitting results for TR-MOKE (left), TR-R (middle), and extracted temperatures (right) for *F*_P_ ranging from 1.7 to 23.1 mJ cm^-2^, as determined from the conventional 3TM. Although the TR-MOKE signal is well fitted, the fitting to the TR-R data exhibits a clear discrepancy from the experimental data. The experimental data not only show the delay of the minimum position, but also reveal the opposite trend in the case of higher fluences. In particular, the observed delay increases as *F*_P_ increases. The behavior of extracted temperatures shows a typical behavior for that case of the electron being heated first and the spin excitation rapidly following, while the lattice is slowly heated until they reach equilibrium. Nevertheless, the simple conventional 3TM remains incapable of fitting both TR-MOKE and TR-R.

**
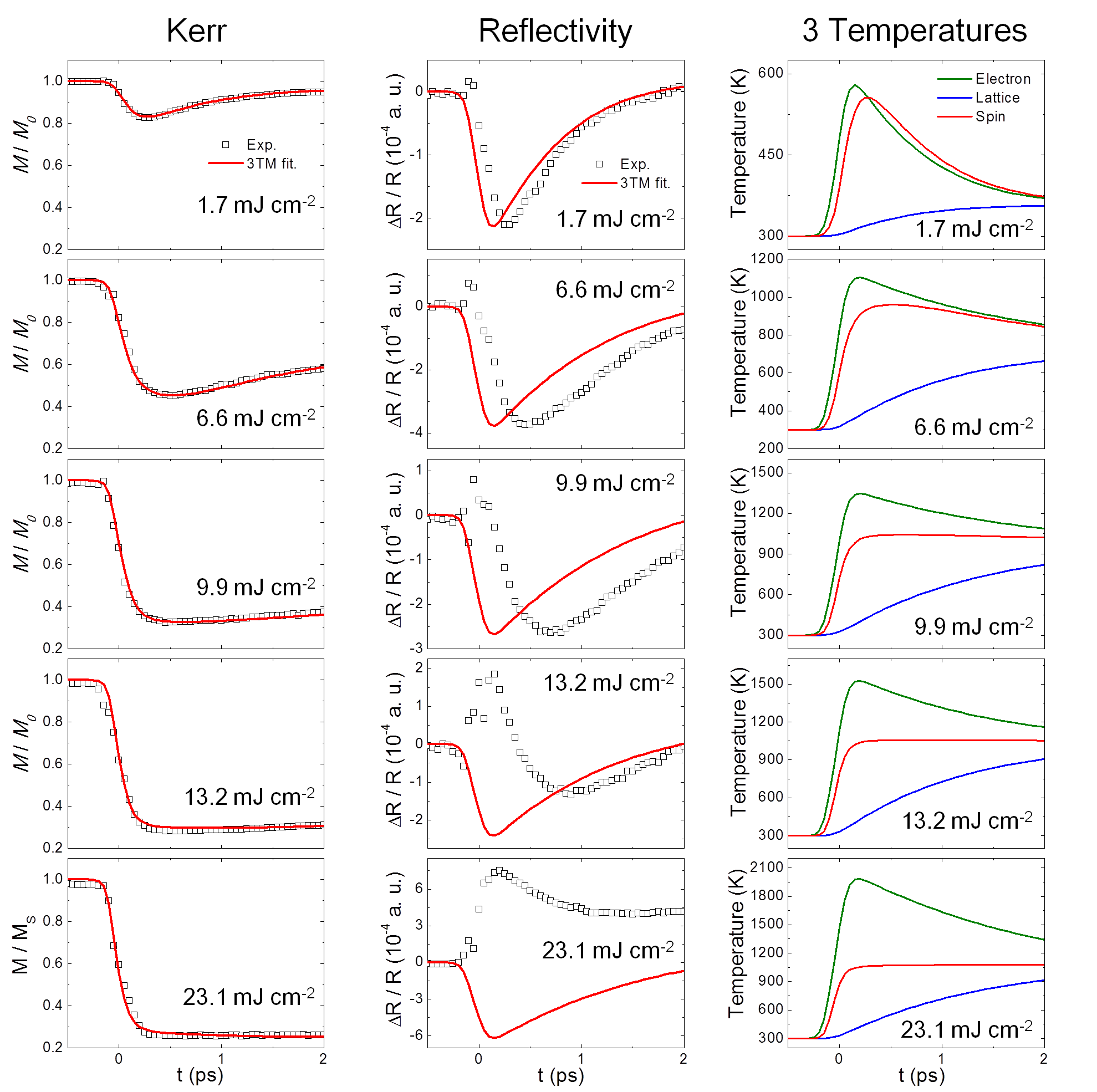
**

**Figure S1.** Best fitting using the conventional 3TM.

The initial positive contribution to the reflectivity in the case of high fluences has been reported by C.-K. Sun *et al*, where the phenomena were explained based on the excitation of non-thermal electrons near the Fermi band by pump laser. To accommodate the effect of non-thermal electrons, E3TM was adopted to fit the TR-MOKE signals and reflectivity with Eq. (1) and (2), respectively. Eq. S(1) was also modified, including a non-thermal electron contribution, to

$\frac{\Delta R}{R}\sim a\Delta T_{e}+b\Delta T_{l}+c\Delta N$ (S3),

where *a*, *b,* and *c* are fitting parameters, *∆T_e_* and *∆T_l_* are the pump-induced change of temperature, and *∆N* is the change of non-thermal electron energy density.

In Fig. S2, the E3TM fitting to TR-R data is shown for *F*_P_ = 1.7, 6.6, and 13.2 mJ cm^-2^, with the contributions from thermal electrons, lattice, and non-thermal electrons being also displayed. The inclusion of the non-thermal electron contribution gave an excellent fit to the TR-R data, and offered a clue as to the delay of the minimum position (dashed box) and the change of the sign of the reflectivity. These are discussed in more detail in the main paper.


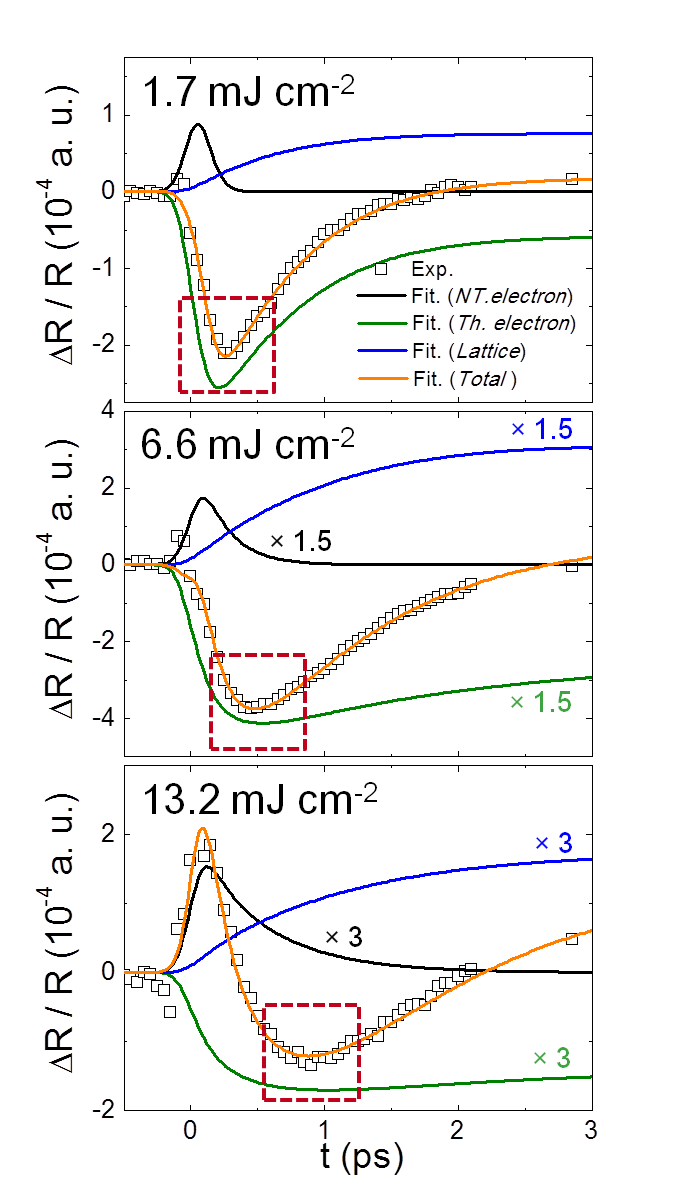


**Figure S2.** Fitting result of E3TM for *F*_P_= 1.7, 6.6, and 13.2 mJ cm^-2^. Experimental data (square open) are fitted by E3TM (orange). Contribution from non-thermal electrons (black), thermal electrons (green), and lattice (blue) are separately shown.

Adopting Manchon's theory, *G_es_* is a function of thermal electrons and spin temperature, and *C_s_* is a function of spin temperature, as follows:

$G_{es}\left[T_{e},T_{s}\right]=G_{es0}\frac{\left(M[T_{s}]\right)^{3}\left(G_{2}\left[\frac{T_{e}}{DT_{C}}\right]-G_{2}\left[\frac{T_{s}}{DT_{C}}\right]\right)}{T_{e}-T_{s}}$ (S4),

 (S5),

$C_{s}[T_{s}]=\left(C_{s0}\times ln\frac{1+(T_{s}/T_{c})^{3}}{1-(T_{s}/T_{c})^{3}}\right)+C_{0}$ (S6),

where *G_es0_* and *C_0_* are values of *G_es_* and *C_s_* at zero temperature,  *a_lat_* is a lattice constant, *S* = 3/2 is a quantum spin number, *V* a unit cell volume, and *K_l_* a thermal diffusion constant. *T_F_* is the Fermi temperature, chosen to be that of fcc Co (*T_F_* = 16.87 *Ry*/*k_B_*). *D* = *SM*[*T_s_*] *q_m_*^2^ *a*^2^, where *q_m_* is magnon wave number *q_m_* = *k_F_* = (6π^2^)^1/3^/ *a_lat_*, and *k_F_* is Fermi wave number. *A_ex0_* is the exchange stiffness at 0 K so that 2*a_lat_ A_ex0_* becomes the exchange coupling constant *J_ex_*. In Eq. S4, the Debye function of *G_n_*[*x*] is defined as

$G_{n}[x]=x^{n+1}\int_{0}^{1/x} \frac{t^{n}}{e^{t}-1}dt$ (S7),

where *n* = 2 in the present case.

**S2. Dynamics of *p_s_*[*t*]**

In Fig. S3, the non-thermal electron energy density, *N*, the electron-spin energy interaction coefficient, *G_es_*, and the transferred energy into spin system, *p_s_*[*t*], are plotted for various cases of *F*_P_. The *N* profiles are basically identical to the profiles shown in Fig. 5B. The *G_es_* profile exhibits an initial sharp increase up to the maximum. In the case of 1.7 mJ cm^-2^, *G_es_* reaches the maximum around t ~ 300 fs, and then decreases monotonically. However, in the case of *F*_P_ > 1.7 mJ cm^-2^, *G_es_* reaches the maximum value for t < 300 fs, where the peak time is shifted earlier as *F*_P_ increases. Given that the TR-MOKE data indicate that the maximum demagnetization occurs at t ~ 300 fs, the maximization of *G_es_* before the maximum demagnetization time implies the additional energy transfer to the spin system.

Since *p_s_*[*t*] is the transferred energy from the non-thermal electron to the spin subsystem, it should be proportional to the product of *N* and *G_es_*. The resulting *p_s_*[*t*] profile exhibits a narrow peak region (orange dashed box in Fig. S3) around t ~ 300 fs, regardless of the fluences. The fact that the narrow peak region of *p_s_*[*t*] is kept at t ~ 300 fs is closely related to the fact that the maximum demagnetization time takes place around t ~ 300 fs for all the fluences, as observed in Fig. 2 in the main paper. The wide tail of *p_s_*[*t*] in Fig. S3 is canceled by the sum of the energy interaction of each system, as in Fig. 6 in the main paper.


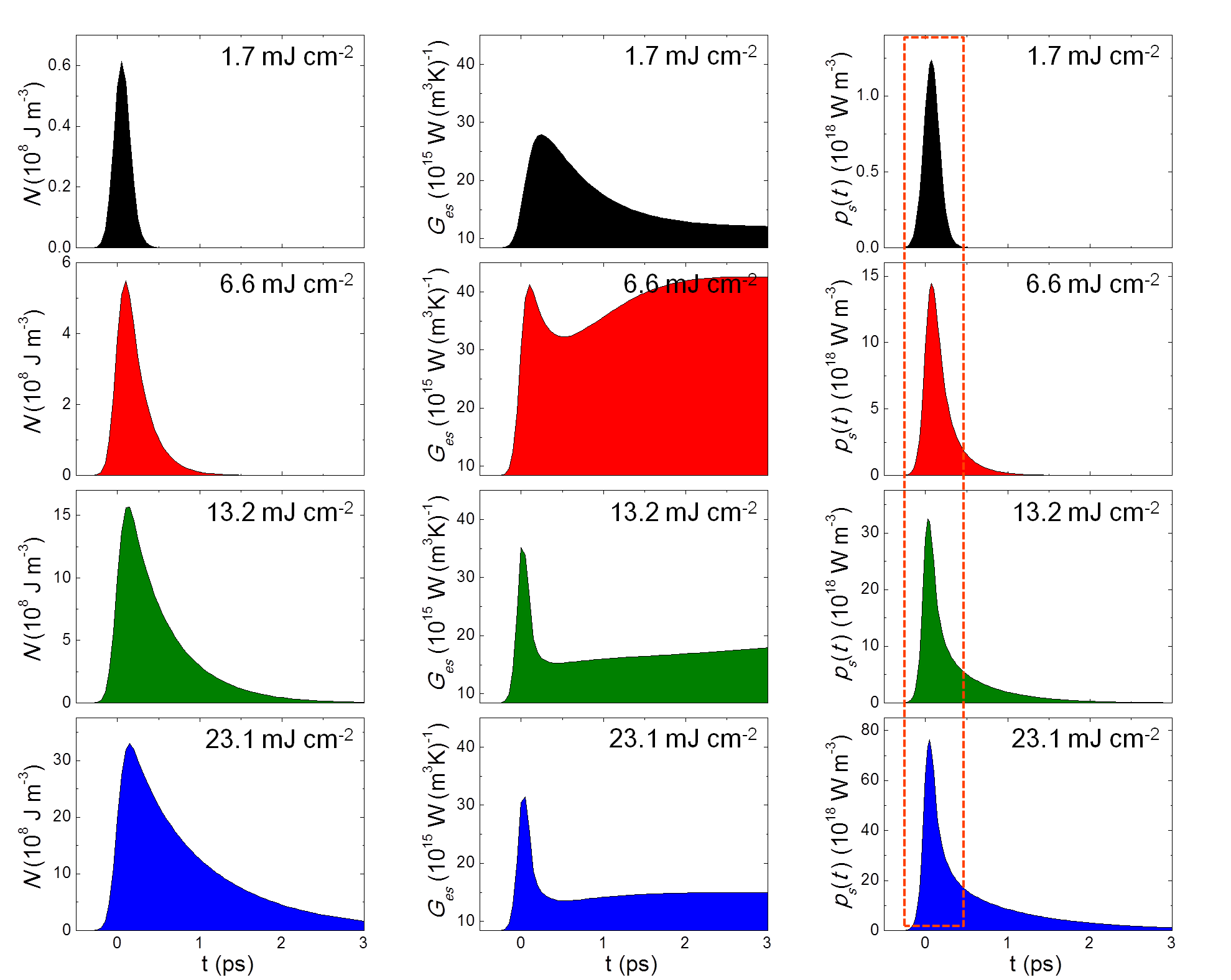


**Figure S3.** Pulse shape of *p_s_*[*t*]. Non-thermal electron energy density *N* (left), electron-spin energy interaction coefficient *G_es_* (middle), and the energy transferred into the spin system *p_s_*[*t*] (right) for *F_P_* = 1.7, 6.6, 13.2, and 23.1 mJ cm^-2^.

**S3. Coherent phonon oscillation in reflectivity.**

We have analyzed the reflectivity over longer timescale for the case of n = 5, as in Figure S4, where we could see the coherent acoustic phonon oscillation behavior^1^. In case of *F*_P_ = 6.6, 13.2, and 23.1 mJ cm^-2^, phonon oscillations with frequencies of 85.2 ~ 89.7 GHz are observed. The first peak of the oscillation is always later than 5 ps as seen in the figure, implying that the strain pulse width is comparable or longer than 5 ps.


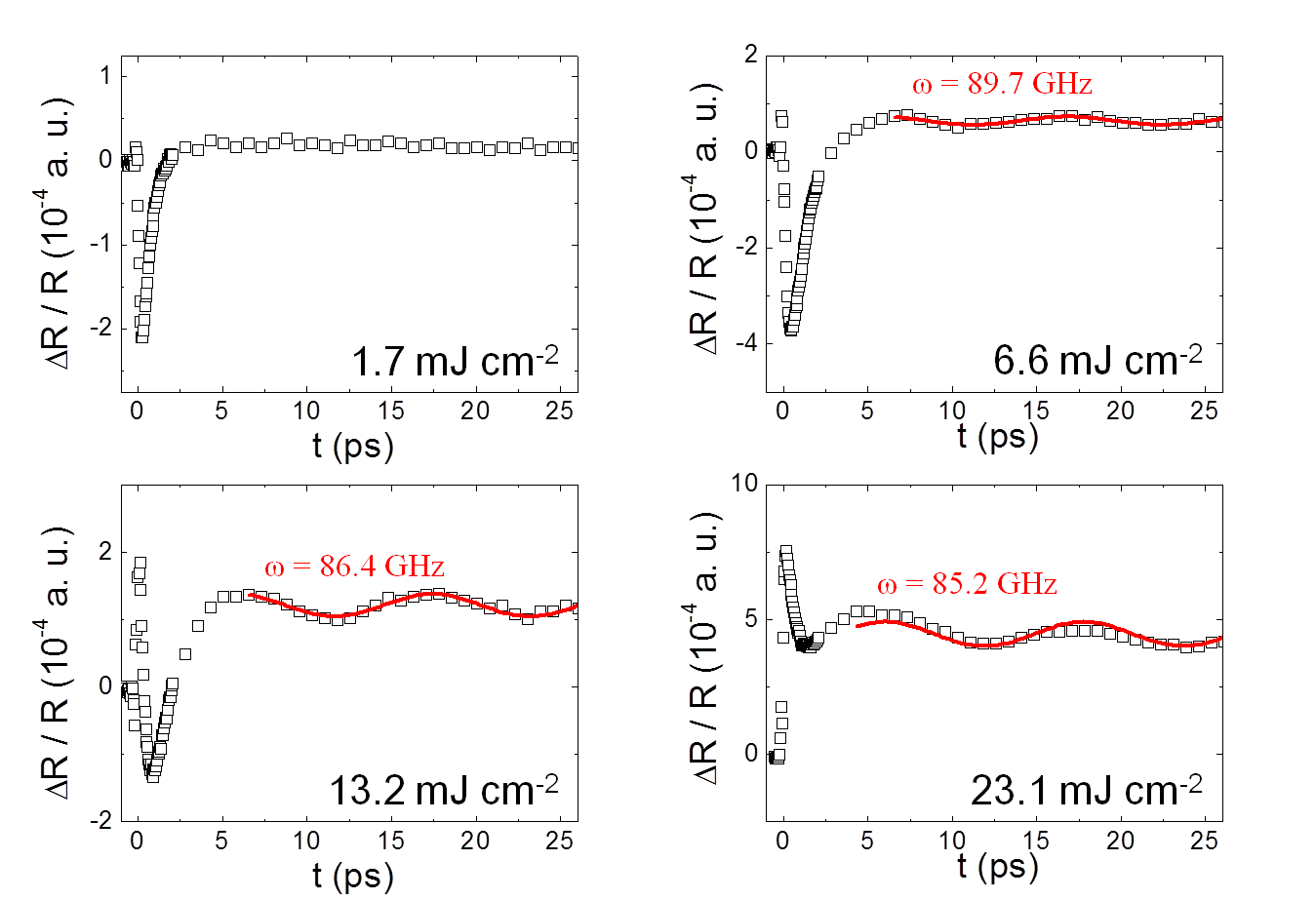


**Figure S4.** TR-reflectivity signal measured during 25 ps for [Co/Pt]_5_ film at various fluences. Red lines are fitting of phonon oscillations.

**References**

1. Matsuda, O. & Wright, O. B. Reflection and transmission of light in multilayers perturbed by picosecond strain pulse propagation. *J. Opt. Soc. Am. B* **19**, 3028–3041 (2002).
